# Humoral and cellular immune response to mRNA SARS-CoV-2 BNT162b2 vaccine in adolescents with rheumatic diseases

**DOI:** 10.1186/s12969-022-00724-4

**Published:** 2022-08-13

**Authors:** Clara Udaondo, Carmen Cámara, Laura Miguel Berenguel, Rosa Alcobendas Rueda, Celia Muñoz Gómez, Claudia Millán Longo, Blanca Díaz – Delgado, Iker Falces-Romero, Mariana Díaz Almirón, Jordi Ochando, Ana Méndez – Echevarría, Agustín Remesal Camba, Cristina Calvo

**Affiliations:** 1 Paediatric Rheumatology Unit, Hospital Infantil La Paz, Paseo de la Castellana 261, 28046 Madrid, Spain; 2 La Paz Institute of Biomedical Research (IdiPAZ), 28046 Madrid, Spain; 3 CIBERINFEC, Instituto de Salud Carlos III (ISCIII), Madrid, Spain; 4 Department of Immunology, Hospital La Paz, 28046 Madrid, Spain; 5 Microbiology and Parasitology Department, Hospital La Paz, 28046 Madrid, Spain; 6 Biostatistics, Hospital La Paz, 28046 Madrid, Spain; 7 National Microbiology Centre, Instituto de Salud Carlos III, 28220 Madrid, Spain; 8 Paediatric and Infectious Diseases Department, Hospital La Paz, 28046 Madrid, Spain; 9 Paediatric Translational Network in Infectious Diseases (RITIP), Madrid, Spain

**Keywords:** Covid-19, mRNA vaccination, Methotrexate, TNF inhibitors, Children, Juvenile Idiopathic Arthritis

## Abstract

**Background:**

Data about safety and efficacy of the mRNA SARS-CoV-2 vaccine in adolescents with rheumatic diseases (RD) is scarce and whether these patients generate a sufficient immune response to the vaccine remains an outstanding question.

**Objective:**

To evaluate safety and humoral and cellular immunity of the BNT162b2 vaccine in adolescents 12 to 18 years with RD and immunosuppressive treatment compared with a healthy control group.

**Methods:**

Adolescents from 12 to 18 years with RD followed at Hospital La Paz in Madrid (*n* = 40) receiving the BNT162b2 mRNA vaccination were assessed 3 weeks after complete vaccination. Healthy adolescents served as controls (*n* = 24). Humoral response was measured by IgG antiSpike antibodies, and cellular response by the quantity of IFN-γ and IL-2 present in whole blood stimulated with SARS-CoV-2 Spike and M proteins.

**Results:**

There were no differences in spike-specific humoral or cellular response between groups (median IFN-γ response to S specific protein; 528.80 pg/ml in controls vs. 398.44 in RD patients, p 0.78, and median IL-2 response in controls: 635.68 pg/ml vs. 497.30 in RD patients, p 0.22. The most frequent diagnosis was juvenile idiopathic arthritis (26/40, 65%) followed by Lupus (6/40, 15%). 60% of cases (23/40) received TNF inhibitors and 35% (14/40) methotrexate. 40% of patients (26/64) had previous SARS-CoV-2 infection, 9 in the control group and 17 in the RD patients without differences. Of note, 70% of infections were asymptomatic. A higher IFN-γ production was found in COVID-19 recovered individuals than in naive subjects in both groups (controls: median 859 pg/ml in recovered patients vs. 450 in naïve p 0.017, and RD patients: 850 in recovered vs. 278 in naïve p 0.024). No serious adverse events or flares were reported following vaccination.

**Conclusions:**

We conclude that standard of care treatment for adolescents with RD including TNF inhibitors and methotrexate did not affect the humoral and the cellular immunity to BNT162b2 mRNA vaccination compared to a healthy control group. The previous contact with SARS-CoV-2 was the most relevant factor in the immune response.

## Introduction

Patients with rheumatic diseases (RD) are considered a high-risk group for COVID-19 because their inherent immune dysregulation as well as the additional disruption that comes from their biological treatment. Consequently, RD patients are included as a priority group within national vaccination strategies for COVID-19 [1]. Several international organizations and national health authorities have developed guidelines for COVID-19 vaccination, including high-risk populations such as patients with Immune Mediated Inflammatory Diseases (IMID) [2–5]. Patients with IMID include subjects with rheumatic diseases (RD) that are treated with immunosuppressive and biologic treatments, such as anti-TNF, which could potentially interfere with the vaccine efficacy [6]. Consistent with this hypothesis, recent studies in adult patients have demonstrated that some biologic drugs, such as rituximab or methotrexate, may decrease immunogenicity of COVID-19 vaccination [5–7].

The BNT162b2 mRNA coronavirus disease 2019 (COVID-19) vaccine was recommended for adolescents aged 12 years and older by the European Medicines Agency (EMA) on May 28, 2021 after evaluation of the results of the clinical trial in terms of efficacy, immune response and safety [8]. Data about safety and efficacy of the mRNA SARS-CoV-2 vaccine in adolescents with RD is scarce and whether these patients generate a sufficient immune response to the vaccine, because the possible interference of biological treatments on cytokine signalling necessary for antigen-specific T cells activation, remains an outstanding question.

This study aimed to evaluate the humoral and the cellular immune response against the BNT162b2 mRNA vaccine in adolescents with RD receiving biologic treatments ranging from 12 to 18 years and compared the results with a healthy control group of the same age.

## Patients and methods

This prospective observational exploratory study was conducted at the Paediatric Rheumatology Unit of Hospital La Paz, Madrid, from August to December 2021. The study protocol was reviewed and approved by Hospital La Paz Clinical Research Ethics Committee (CEIm– PI 4926). Parents and participants signed and informed consent at inclusion. Participation in this study did not change the vaccine indication or treatment indicated by clinical practice.

### Objectives

The primary endpoint was to measure spike-specific humoral and cellular immunity of the BNT162b2 mRNA vaccine in adolescents (12- 18 years old) with rheumatic diseases (RD) under biologic treatment compared with healthy controls of the same age, measured 3 weeks after the complete vaccination regimen. According to vaccination strategy of the Spanish Ministry of Health in force during the study, complete vaccination schedule was defined by one-dose of the mRNA vaccine in those individuals with previous COVID-19 infection (recovered) or after a two-dose regimen of the mRNA vaccine administered 3 weeks apart otherwise (naïve) [9].

Secondary objectives were to investigate the difference in the immune response after vaccination between naïve and COVID-19 recovered individuals in both groups, and to analyse the RD group looking for differences on the immune response to the vaccine depending on the type of immunosuppressive treatments.

### Study population

Inclusion criteria for all groups was having received the full SARS-CoV-2 vaccination schedule. Children aged between 12 to 18 years with rheumatic diseases (RD) were recruited into the study according to the following diagnosis: Juvenile Idiopathic Arthritis (JIA, ILAR criteria), juvenile onset Systemic Lupus Erythematosus (joSLE, SLICC criteria), Uveitis, Juvenile Systemic Sclerosis (jSSc, PRES/ACR/EULAR criteria), Juvenile Dermatomyositis (JDM; Bohan and Peter criteria), Mixed Connective Tissue Disease (MCTD, Sharp criteria), Behçet’s disease (International Study Group criteria), Crohn’s disease, A20 Haploinsufficiency (HA20). Almost all patients were under immunosuppressive treatment and were instructed to continue all medications during the vaccination period, and no drug discontinuation after vaccination was recommended.

The control group included children aged between 12 to 18 years, receiving vaccination against COVID-19 in our hospital. Exclusion criteria for controls were history or suspicion of chronic disease affecting the immune response and immunosuppressive treatment including corticosteroids.

### Immunogenicity of the vaccine

Peripheral blood samples were obtained from healthy naive and COVID-19 recovered RD patients and donors three weeks (± 4 days) after full vaccination. The characterization of host antibodies response was evaluated by detection of anti-S total antibodies using chemiluminescence (CLIA) (Atellica® IM SARS-CoV-2 Total COV2T, Siemens Healthineers). A value above 1 index was considered as positive, according to the manufacturer’s instruction. The maximum value of IgG response was over 10 index. To assess the specific T cell response, we measured IFN-γ and IL-2 present in whole blood stimulated with SARS-CoV-2 proteins: M protein and pools of proteins S/S1/S + as recently described [10, 11].

### Previous COVID-19 infection

All participants were asked about their history of COVID-19 infection, confirmed by a positive PCR or antigen test result. In order to assess asymptomatic COVID-19 infection, quantity of IFN-γ and IL2 response to M specific protein (not stimulated by the BNT162b2 SARS-CoV-2 vaccine) was performed in all patients.

### Safety of the vaccine

The participants were contacted after the completion of the last dose to complete a questionnaire regarding adverse events. Serious adverse events included those who required admission into the emergency unit, hospitalization or any kind of sequelae.

### Clinical assessment of children with rheumatic diseases

Medical history and the use of medications were recorded. Data regarding disease activity before vaccination were retrieved from patients’ medical records within 3 months before vaccination, using the physician’s global assessment visual analogue scale of 0–10 mm. A visual analogue scale (VASc) of 0 was considered as inactive disease.

### Statistical analysis

Quantitative data were expressed using mean and standard deviation, or median and interquartile range, where appropriate. Qualitative data were expressed using absolute frequencies and percentages. The univariate comparative analysis, in the case of qualitative variables, was carried out using Pearson’s Chi-square test or Fisher’s exact test. In the case of quantitative variables, comparisons were made by non-parametric tests due to the small sample size. When the continuous variable was analysed by one factor, the Mann–Whitney test was used. *P* values below the 0.05 threshold were considered statistically significant. All tests were considered bilateral. The statistical software used was SAS Enterprise Guide 8.2. (Cary NC, SAS Institute Inc).

## Results

### Demographics

A total of 64 patients were recruited in the study, including 40 adolescents with RD and 24 healthy adolescents in the control group. No differences were found in age and sex between both groups (53% female, median 14 years, range 12–16). The most frequent diagnosis of patients with RD was juvenile idiopathic arthritis (JIA, 26/40 65%) followed by connective tissue diseases (8/40, 20%) including juvenile onset Systemic Lupus Erythematosus (joSLE, 6/40), juvenile Dermatomyositis (JDM 1/40) and juvenile Systemic Sclerosis (jSSc 1/40). All but 5 patients (12.5%) (4 JIA, 1 joSLE) were under immunosuppressive treatment. A total of 24/40 patients (60%) received biologic therapy, the most frequent being TNF inhibitors (11 adalimumab, 9 etanercept, 3 infliximab). Other treatments received were mycophenolate mofetil (5/40), baricitinib (5/40) and cyclosporine (1/40). A total of 14 patients (35%) received methotrexate between 10 and 15 mg/m^2^/weekly combined with other treatment. None of the patients received systemic corticosteroids above 20 mg/day or above 0.5 mg/kg/day at the time of inclusion. Demographic characteristics, diagnosis and treatments of patients are shown in Table 1.

**Table 1:** Demographic characteristics of patients with rheumatic diseases and controls

	**Sum**	**Controls**	**Adolescents with RD**	*p*
n	64	24	40	
Age				
(median, range)	14 (12 – 16)	13 (12 – 14)	14 (13 – 16)	*0.07*
Sex				
Female n (%)	34 (53%)	12 (50%)	22 (55%)	*0.79*
**Description of patients with rheumatic diseases:**				
**Diagnosis n (%)**				
JIA			26 (65%)	
joSLE			6 (15%)	
Uveitis			3 (7.5%)	
JDM			1 (2.5%)	
Juvenile Systemic sclerosis			1 (2.5%)	
Crohn´s disease			1 (2.5%)	
Behçet`s disease			1 (2.5%)	
HA20			1 (2.5%)	
**Treatment n (%)**				
Adalimumab			11 (27.5%)	
Etanercept			9 (22.5%)	
Infliximab			3 (7.5%)	
Mycophenolate			5 (12.5%)	
Baricitinib			5 (12.5%)	
Tocilizumab			1 (2.5%)	
Cyclosporine			1 (2.5%)	
None of the above			5 (12.5%)	
Methotrexate			14 (35%)	
**Disease Activity n (%)**				
Inactive disease *(VASc* = *0)*			26 (65%)	
Active disease *(VASc* > *0)*			14 (35%)	

*JIA* Juvenile Idiopathic Arthritis, *joSLE* Juvenile Onset Systemic Lupus Erythematosus, *JDM* Juvenile Dermatomyositis, *HA20* Haploinsufficiency A20, *VASc* Visual Analogue Scale – clinician

### Previous Covid-19 infection

Eight patients reported previous COVID-19 infection, 4 in each group. When performing the specific cellular response to SARS-CoV-2 M protein (not stimulated by the vaccine), we found that 24 patients (42%), 8 controls (34%) and 16 RD patients (40%), had a positive response, meaning previous SARS-CoV-2 infection, with no differences between both groups. Out of these 24 patients, 16/24 (66%) were unaware that they had been infected. Two patients (1 in each group) reported previous positive PCR for SARS-CoV-2, but had negative IFN-γ response to M peptide stimulation. Overall, 70% (18/26) of all recovered patients had an asymptomatic infection, 5/9 in the control group (55%) and 13/17 in the RD group (76%) Table 2.

**Table 2:** Previous COVID-19 infection

	**Sum** ***n* = 64**	**Controls** ***n* = 24**	**Adolescents with RD *n* = 40**	*p*
Reported COVID-19 infection *SARS-CoV-2* + *PCR* n (%)	8 (13%)	4 (18%)	4 (10%)	
Demonstrated COVID-19 infection *IFN*-γ *M cellular response* n (%)	24 (42%)	8 (34%)	16 (40%)	*0.78*
SARS-CoV-2 + PCR with negative IFN-γ M response (n)	2	1	1	
Global SARS-CoV-2 infection	26/64 (40%)	9/24 (37%)	17/40 (42%)	
Previously unknown SARS-CoV-2 infection n (%)	18/26 (70%)	5/9 (55%)	13/17 (76%)	

### Humoral and cellular response to SARS-CoV-2 vaccine

Evaluation of the spike-specific T cell responses three weeks after the complete vaccination regimen showed no significant differences in spike-specific IFN-γ or IL-2 production between RD patients and the control group (median IFN-γ response to S specific protein; 528.80 pg/ml in controls vs. 398.44 in RD patients; p 0.78, and median IL-2 response in controls: 635.68 pg/ml vs. 497.30 in RD patients; p 0.22). Results are shown in Table 3.

**Table 3:** Humoral and Cellular response against the mRNA BNT162b2 vaccine in adolescents with RD and controls

	**Controls** ***n* = 24**			**Adolescents with RD *n* = 40**		***P***
IFN-γ (pg/mL, median, IQR)	529 (288 – 935)			398 (209 – 1025)		*0.78*
IL-2 (pg/mL, median, IQR)	636 (265 – 834)			497 (339 – 611)		*0.22*
SARS-CoV-2 IgG antibodies > 10 index (%)	23/24 (96%)			38/40 (95%)		
	Naive	COVID-19 recovered	*p*	Naive	COVID-19 recovered	*p*
IFN-γ (pg/mL, median, IQR)	450 (229 – 708)	859 (412 – 1452)	*0.017*	278 (202 – 784)	849 (342 – 1526)	*0.024*
IL-2 (pg/mL, median, IQR)	398 (255 – 571)	592 (490 – 705)	*0.036*	632 (255 – 794)	675 (364 – 1048)	*0.28*
	Naïve controls (*n* = 15)			Naïve adolescents with RD (*n* = 23)		*p*
IFN-γ (pg/mL, median, IQR)	450 (229 – 708)			278 (202 – 784)		*0.55*
IL-2 (pg/mL, median, IQR)	398 (255 – 571)			632 (255 – 794)		*0.23*
	Controls (*n* = 24)			Adolescents with RD (JIA + SLE) (*n* = 32)		
IFN-γ (pg/mL, median, IQR)	529 (288 – 935)			451 (216 – 1022)		*0.89*
IL-2 (pg/mL, median, IQR)	636 (265 – 834)			653 ( 361 – 814)		*0.13*

*IQR* Interquartile range, *JIA* Juvenile Idiopathic Arthritis, *joSLE* Juvenile Onset Systemic Lupus Erythematosus *RD* Rheumatic diseases

Considering previous COVID-19 infection, the spike peptide pool induced a higher IFN-γ production in COVID-19 recovered individuals than in naïve subjects in both groups (controls: median 859 pg/ml in recovered patients vs. 445 in naïve; p 0.017, and RD patients: 849 in recovered vs. 278 in naïve; p 0.024). We observed a similar trend in spike-specific IL-2 production in controls (median 591 pg/ml in recovered individuals vs. 398 in naïve; p 0.036) but not in RD patients (median 675 pg/ml in recovered individuals vs. 632 in naïve; p 0.28).

To try to compare more homogeneous groups, we performed a sensitivity analysis comparing the spike-specific T cell response between naïve RD patients and controls, eliminating those with previous COVID-19 infection (evidenced either by positive PCR or specific cellular response to SARS-CoV-2 M protein). We found no differences among these groups**.** We also compared a reduced RD patient group including only those diagnosed with JIA or joSLE with the control group, with no differences (Table 3).

Regarding humoral response, at the time of the study 61/64 patients (95%) had anti-S IgG titres greater than 10 index, which is considered the maximum value. No differences in humoral response were found between RD patients and controls, or between COVID-19 naive and recovered patients.

### Immunogenicity and biologic treatment

Comparing the largest therapeutic group receiving TNF inhibitors (*n* = 23) with the rest of the RD patients (*n* = 17), we found that those subjects mount a stronger IFN-γ and IL-2 response in comparison with the rest of the RD patients (median IFN-γ response to S specific protein; 761 pg/ml in TNF vs. 298 in non TNF RD patients; p 0.012, and median IL-2 response in TNF: 754 pg/ml vs. 343 in non TNF treated RD patients; p 0.014). Of note, of the 17 COVID-recovered RD patients, 12/17 were receiving antiTNF treatment and 5/17 were receiving other treatments. No differences were found between diagnosis (JIA vs. other diagnosis), treatment with methotrexate vs. other treatments, or disease activity and immunogenicity of BNT162b2 COVID-19 vaccine in RD patients.

### Safety of BNT162b2 vaccine

Side effects were comparable in both groups. Local pain or swelling were the most common side effects for both groups (54% vs. 50% in controls and RD patients respectively), followed by mild systemic side effects such as low-grade fever (17% vs. 20%) or asthenia (8% vs. 18%). An additional side effect reported by one participant was menstrual disorder. However, approximately one third of patients did not have side effects. We did not observe any inflammatory arthritis flares in JIA patients in the context of either vaccination time points. No severe side effects were reported (Table 4).

**Table 4:** Adverse events after SARS-CoV-2 vaccination

	**Controls**	**Adolescents with RD**
AE after 1º dose		
Local	13 (54%)	20 (50%)
None	7 (30%)	11 (28%)
Asthenia	1 (4%)	2 (5%)
Low grade fever	2 (8%)	2 (5%)
Not reported	1 (4%)	5 (12%)
AE after 2º dose n (%)		
Low grade fever	4 (17%)	8 (20%)
Local	2 (8%)	8 (20%)
Asthenia	2 (8%)	7 (18%)
None	1 (4%)	3 (8%)
Menstrual disorder	0	1 (2%)
Not reported	11 (46%)	9 (22%)
Not applicable	4 (17%)	4 (10%)

## Discussion

The results of this preliminary study show that adolescents with RD receiving the BNT162b2 mRNA COVID-19 vaccine mount strong immune humoral and cellular responses, no different than a healthy control group, despite their standard of care treatments. The vaccine was safe and effective in this group of patients, arguing in favour of continuing biological treatment during SARS-CoV-2 vaccination.

Adolescents with RD treated with immunosuppressive agents observed a robust humoral and spike-specific T cell response unlike previous studies in adults [6, 7]. Many immunosuppressive drugs have been associated with reduced antibody titres and neutralization activity following COVID-19 vaccination in adult patients receiving B-cell–depleting therapies and methotrexate (MTX) [12–14]. Therefore, the last expert guidelines recommend a modification of the treatment in some cases, such as withhold methotrexate for 1 week after vaccination [2–5]. Our cohort included 14 patients treated with MTX and no differences on humoral or T cell responses were observed on these MTX treated patients without interrupting treatment. However, our small sample size may have limited the statistical power needed to demonstrate differences across the two groups. Future studies with larger sample sizes are needed to corroborate our results. It is important to note that our study group did not include rituximab, related to a decrease in humoral response in adults with IMID [7, 12]. Recommendations for vaccination against SARS-CoV-2 in children with RD are based on adult population studies, although there is no clear consensus about treatment modifications regarding mRNA vaccines [15]. In our study, children were instructed to continue their treatment, based on the limited data on this population.

In fact, very little is known about the immune response in adolescents with rheumatic diseases, with recent publications on the safety and tolerability of the vaccine [16, 17]. Similar findings about safety and tolerability were obtained in this study, with most adverse effects being mild and no serious adverse effects reported. Further studies are needed to evaluate the immune response and the immunogenicity of the actual vaccine schedule (two dose for naïve patients and one dose for recovered patients), as well as the need of a booster dose in this group of patients. Preliminary studies of response to mRNA SARS-CoV-2 vaccination in other groups of immunosuppressed adolescents have been done. Crane et al. studied humoral response to mRNA SARS-CoV-2 vaccination in 25 adolescent kidney transplant recipients (receiving calcineurin inhibitors, some in combination with mycophenolate), with a lower response than that of the general population, but similar to that previously described in adult solid organ transplant patients [18]. To the best of our knowledge, cellular response in this patient group has not been studied before.

Interestingly, significant data in the immune response to vaccination on adolescents with RD was obtained when prior exposure to SARS-CoV-2 was valuated, which has been previously described in immunocompetent patients [6, 10, 19] showing the superiority of the hybrid immunity over the vaccine-generated immunity alone [20]. We observed higher levels of IFN-γ secretion in COVID-19 recovered RD patients and controls, but not in naïve individuals. This is consistent with our recent report, which demonstrated that pre-exposed individuals mount stronger IFN gamma responses [21]. Therefore, a precise measurement of cellular responses underlying virus protection represents an important parameter of immune defense [22], even when SARS-CoV-2-specific IgG and neutralizing antibody quantification are being used as clinical endpoints to determine immune protection [21]. In this respect, our data indicate that the humoral immunity does not increase following administration of the BNT162b2 mRNA vaccine. Other studies have also highlighted that humoral immune measurements do not always correlate with the magnitude of T cell response [23], which is evident in non-seroconverters or low-neutralizer patients [24]. Indeed, different groups have demonstrated the presence of SARS-CoV-2-specific T cells in the absence of antibodies [10, 25] and that antibodies and T cell responses are vastly independent in terms of their magnitude and persistence during the memory phase of the immune response [26, 27].

The main group of our cohort under biological treatments received TNF inhibitors (11 adalimumab, 9 etanercept and 3 infliximab). Previous data suggest that patients under this therapy may mount less robust immune responses than those seen in healthy controls [28, 29]. However, we found that those adolescents receiving TNF inhibitors mount a stronger IFN-γ and IL-2 response in comparison with the rest of the RD cohort. A reasonable explanation could be the prior exposure to SARS-CoV-2, as TNF patients represent 70% (12/17) of the COVID-19 recovered group in RD patients, so the stronger cellular response is likely to be related to this fact. From our study, we believe that it is not necessary to discontinue immunosuppressive treatment in adolescents with RD due to SARS-CoV-2 vaccine administration, since no adverse effects and no decrease in the immune response to the vaccine was detected following vaccination.

Finally, it should be noted that we found a high number of RD patients with previous asymptomatic infection, in comparison with the control group (76% in RD patients compared to 55% in the control group). This data suggests that children with RD under immunosuppressive therapy do not represent a high-risk group for severe forms of COVID-19. The possibility of a predisposing role for COVID-19 in TNF inhibitors should be taken into account, as they represent the majority of the previous asymptomatic COVID-19 patients, despite the results did not achieve statistical significance. Another hypothesis could be the role of the immunosuppressive therapy in the development of symptoms of COVID-19. This finding warrants further investigation, because results could have been affected by timing, treatment, or other epidemiological factors. Overall, our results suggest that adolescents with RD receiving immunosuppressive treatment are not a risk group for severe COVID-19, but more likely to develop an asymptomatic infection. Although they have been included in the COVID-19 high risk group [1], considering that RD patients and healthy controls exhibit a similar immune response to the vaccine, and considering the high percentage of asymptomatic infection in RD patients, treatment modifications may not be needed it may be not necessary to administer booster doses in this group.

To the best of our knowledge, this is the first work to assess humoral and cellular response of mRNA vaccine in a population of adolescents with rheumatic diseases under immunosuppressive treatment. Despite study limitations such as small sample size, single-centre experience, and single blood collection of individuals, this study brings a reassuring message to RD patients and healthcare practitioners. Larger studies with more detailed measurements including cell-mediated responses over time are required to assess immune responses and the effects of medications. In the meantime, our results support the consensus recommendation for RD patients to receive mRNA COVID-19 vaccine and support the idea that their immune response to the vaccine is strong so it may not be necessary to discontinue their treatments.

## Conclusion

In this preliminary study, we conclude that standard of care treatment for adolescents with RD, including anti TNF and MTX, does not affect the humoral and the cellular immunity to BNT162b2 mRNA vaccination. This argues in favour not to discontinue immunosuppressive treatment in these patients because it does not seem to negatively impact the immune response and no important adverse effects were observed. Considering that RD patients and healthy controls exhibit a similar immune response to the vaccine, it may not be necessary to administer booster doses in these adolescents despite being a COVID-19 high-risk group. The previous contact with SARS-CoV-2 was the most relevant factor in the immune response.

## Data Availability

The datasets used and/or analysed during the current study are available from the corresponding author on reasonable request.

## Abbreviations

HA20: Haploinsufficiency A20
IFN: Interferon
IMID: Immune Mediated Inflammatory Diseases
IQR: Interquartile range
JDM: Juvenile Dermatomyositis
JIA: Juvenile Idiopathic Arthritis
joSLE: Juvenile Onset Systemic Lupus Erythematosus
MCTD: Mixed Connective Tissue Disease
MTX: Methotrexate
RD: Rheumatic Diseases
TNF: Tumour Necrosis Factor
VASc: Visual Analogue Scale – clinician
